# Identifying Misinformation About Unproven Cancer Treatments on Social Media Using User-Friendly Linguistic Characteristics: Content Analysis

**DOI:** 10.2196/62703

**Published:** 2025-02-12

**Authors:** Ilona Fridman, Dahlia Boyles, Ria Chheda, Carrie Baldwin-SoRelle, Angela B Smith, Jennifer Elston Lafata

**Affiliations:** 1 Lineberger Comprehensive Cancer Center University of North Carolina Chapel Hill, NC United States; 2 Department of Communication University of North Carolina Chapel Hill, NC United States; 3 Computer Science Department University of North Carolina Chapel Hill, NC United States; 4 Health Sciences Library University of North Carolina Chapel Hill, NC United States; 5 Eshelman School of Pharmacy University of North Carolina Chapel Hill, NC United States

**Keywords:** linguistic characteristics, linguistic features, cancer, Linguistic Inquiry and Word Count, misinformation, X, Twitter, cancer, alternative therapy, oncology, social media, natural language processing, machine learning, synthesis, review methodology, search, literature review

## Abstract

**Background:**

Health misinformation, prevalent in social media, poses a significant threat to individuals, particularly those dealing with serious illnesses such as cancer. The current recommendations for users on how to avoid cancer misinformation are challenging because they require users to have research skills.

**Objective:**

This study addresses this problem by identifying user-friendly characteristics of misinformation that could be easily observed by users to help them flag misinformation on social media.

**Methods:**

Using a structured review of the literature on algorithmic misinformation detection across political, social, and computer science, we assembled linguistic characteristics associated with misinformation. We then collected datasets by mining X (previously known as Twitter) posts using keywords related to unproven cancer therapies and cancer center usernames. This search, coupled with manual labeling, allowed us to create a dataset with misinformation and 2 control datasets. We used natural language processing to model linguistic characteristics within these datasets. Two experiments with 2 control datasets used predictive modeling and Lasso regression to evaluate the effectiveness of linguistic characteristics in identifying misinformation.

**Results:**

User-friendly linguistic characteristics were extracted from 88 papers. The short-listed characteristics did not yield optimal results in the first experiment but predicted misinformation with an accuracy of 73% in the second experiment, in which posts with misinformation were compared with posts from health care systems. The linguistic characteristics that consistently negatively predicted misinformation included tentative language, location, URLs, and hashtags, while numbers, absolute language, and certainty expressions consistently predicted misinformation positively.

**Conclusions:**

This analysis resulted in user-friendly recommendations, such as exercising caution when encountering social media posts featuring unwavering assurances or specific numbers lacking references. Future studies should test the efficacy of the recommendations among information users.

## Introduction

Approximately 16% of people reported using social media to inform their medical decisions [1]. This percentage, based on estimates from the National Cancer Center, equates to 37 million adults in the United States. A recent systematic review estimated that up to 40% of health-related social media posts contain misinformation [2]. Misinformation could cause more harm to individuals with serious conditions such as cancer. Patients who believe in misinformation and use unproven therapies in parallel or in place of cancer treatment tend to be less adherent to evidence-based treatment [3-5]. Moreover, patients with cancer might choose to delay or reject evidence-based treatment and instead pursue unproven and potentially toxic therapies, which, for some patients, results in up to 2.5 times shorter life expectancy [6]. Approximately 30% of cancer-related social media posts on Facebook, Reddit, Pinterest, and X (previously known as Twitter) contain misinformation, and a staggering 77% of these posts have the potential to encourage patients to pursue futile and toxic therapies, resulting in physical, psychological, and logistical burdens [7]. Cancer misinformation persists across various cancer types and is more pervasive in more prevalent cancers. Across various social media platforms, two-thirds of the most shared posts about prostate cancer contain misinformation [8]. Researchers identified misinformation in 59% of posts related to breast cancer prevention and treatment [9] and 30% of posts related to gynecological cancer [10]. When surveyed, 70% of patients with cancer reported encountering misinformation about cancer on social media, with 71% believing that some of this misinformation was accurate [11].

There is a growing need to protect health information users from misinformation, especially those who are affected by serious conditions such as cancer. Multiple recommendations have been developed to assist individuals in their search for reliable health information [12-14]. However, many of the recommendations are complex, as they require individuals to possess a certain level of scientific knowledge and skills. For instance, recommendations frequently suggest taking steps such as identifying authors and their credentials, evaluating potential conflicts of interest, understanding funding sources, and assessing the original sources of scientific information. Considering the time and expertise required, expecting individuals to perform these tasks routinely is unrealistic. Moreover, these guidelines often fall short when it comes to addressing the challenges posed by social media platforms. Those who post may not disclose their real names or sources of findings, which makes some recommended steps not possible.

In this work, our goal is to identify user-friendly recommendations for addressing the high rate of misinformation on social media. We began by exploring literature on the algorithmic detection of misinformation. The algorithmic approach often involves the analysis of linguistic characteristics that differentiate between factual information and misinformation [15]. Linguistic characteristics describe a body of text in an abstract manner regardless of context and may include counts of words and word parts such as nouns, verbs, adjectives, and negations, as well as specific symbols such as URLs, hashtags, and question marks. An additional category of linguistic characteristics includes words associated with the psychological state of an author [16], which includes words related to emotions, expressions of certainty, tentativeness, insight, persuasion, and gratitude. To date, linguistic characteristics have been used by algorithms only. However, some of these characteristics are observable and could be used by individuals when they need to evaluate the credibility of the text. While individuals are unlikely to count words in social media posts regularly, they may routinely note other linguistic characteristics, such as emotions, URLs, and a strong degree of certainty. Linguistic characteristics have been shown to be effective in distinguishing misinformation from factual information across multiple contexts. However, it is unknown (1) whether the linguistic characteristics are effective in cancer-related context and (2) which subset of user-friendly linguistic characteristics could effectively distinguish misinformation. In this work, we identify the linguistic characteristics specific to the context of cancer. These characteristics will be recommended as guidelines for health information users when browsing social media.

## Methods

### Study Design

**Figure 1:**
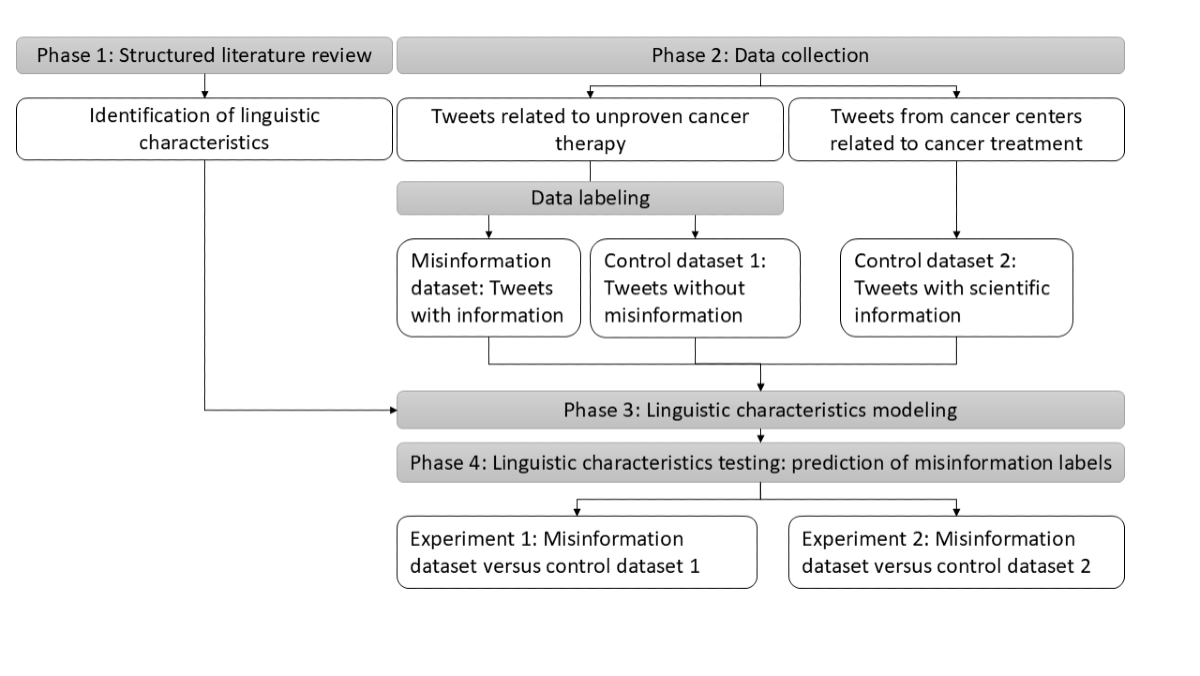
Summary of the study procedures.

The main sequence of study procedures is illustrated in Figure 1 and includes (1) a structured literature review, in which we assemble linguistic characteristics that were used in algorithms for distinguishing factual information and misinformation (phase 1); (2) data collection, which encompasses assembling cancer-related posts using the X application programming interface (API) and labeling them as misinformation and non-misinformation (phase 2); (3) identification of the linguistic characteristics in collected datasets using natural language processing tools (phase 3); and (4) conducting predictive modeling analysis to evaluate the effectiveness of linguistic characteristics in distinguishing social media posts with cancer misinformation (phase 4).

### Ethical Considerations

The study was institutional review board–approved by the University of North Carolina (IRB#21-2861). This was an analysis of publicly available data. As such, participants were not compensated and did not need to provide consent for the study, because the study did not involve any prospective data collection. To protect the confidentiality and anonymity of participants in this secondary data analysis, we reworded reported posts from X.

### Structured Literature Review

To identify linguistic characteristics, we developed a literature review protocol that included the search strategy and keywords. This process was informed by a collaboration with a health sciences librarian (CBS), who suggested an initial set of keywords referenced in several relevant reviews [17-21]. She also created an expanded title, abstract, and keyword search strategies for each of the following concepts: (1) text as a unit of analysis, (2) misinformation, (3) algorithms, (4) internet, and (5) linguistic features or characteristics. After the search was peer reviewed by a second health sciences librarian (CB), 5 databases were searched: ProQuest Central (ProQuest), which includes the arXiv repository; Scopus (Elsevier); IEEE Xplore (Institute of Electrical and Electronics Engineers); ACM Digital Library (Association for Computing Machinery); and Communication & Mass Media Complete (EBSCOhost). The keywords and search strategies are reported in Multimedia Appendix 1. Results were limited to citations published between January 2012 and December 2022. Within databases, results were limited to journal papers, conference proceedings, working papers, and book chapters.

**Figure 2:**
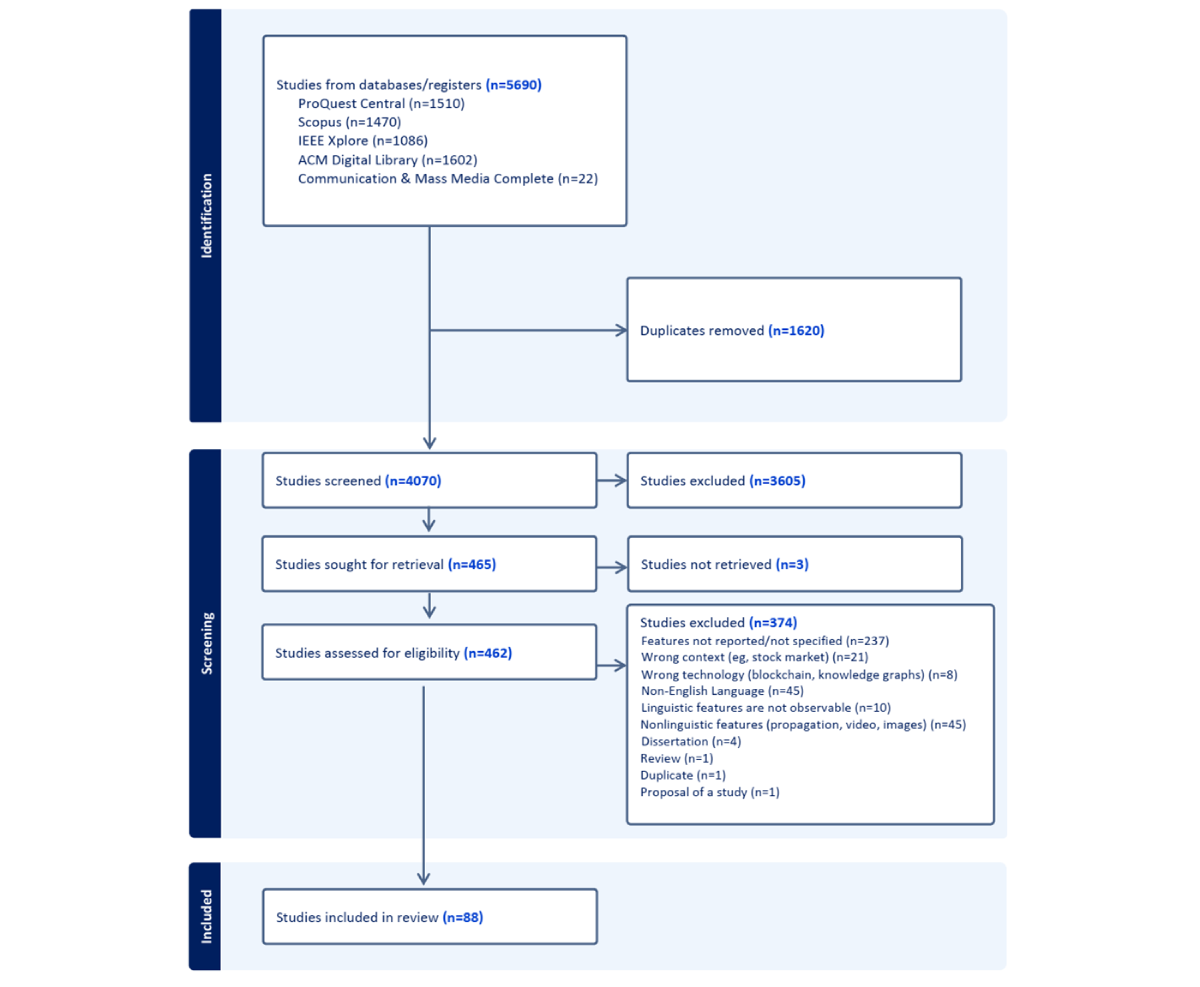
Flowchart of paper identification and extraction.

Two reviewers (IF and DB) independently coded titles and abstracts in Covidence software (Veritas Health Innovation) [22] and resolved conflict in codes during research meetings. Papers were included if they focused on detecting misinformation and contained a “Methods” section describing an approach for algorithmically detecting misinformation (eg, reviews and viewpoints were excluded). Examples of the algorithms included supervised and semisupervised machine learning (eg, Bidirectional Encoder Representations from Transformers [BERT] classification) that was built on linguistic characteristics. Papers were excluded if they did not report specific linguistic characteristics, focused on misinformation in any language other than English, or used human coding but not algorithms. The detailed inclusion-exclusion criteria and PRISMA (Preferred Reporting Items for Systematic Reviews and Meta-Analyses) diagram are reported in Figure 2.

### Identification of Linguistic Characteristics

Upon identifying eligible papers, 2 team members (IF and DB) reviewed the full text and extracted the linguistic characteristics. Around 11% (10/90) of papers underwent double-coding. After reviewers reached an agreement, we continued with single coding. The linguistic characteristics were extracted based on the following criteria: observability, applicability, and generalizability. The observability criterion was related to whether readers could easily observe the linguistic characteristics within the text; for example, positive emotions could be easily observed while morale or cognitive language styles may be difficult to distinguish. The applicability criterion distinguished linguistic characteristics that readers could easily apply while reading the text. For instance, common characteristics such as the number of words required substantial effort from readers to evaluate and, therefore, were deemed nonapplicable. In contrast, readers could easily use citations and hashtags in their post evaluations as the mere presence of these characteristics was determined to be helpful in identifying misinformation. The third criterion, generalizability, was chosen to ensure that linguistic characteristics were not related to a specific context but could be applied across various contexts. Thus, characteristics that were based on specific words such as “COVID-19,” or “cure” were excluded.

### Data Collection: Unproven Therapy

#### Overview

To test how extracted linguistic characteristics could distinguish social media posts from misinformation and factual information, we collected social media posts from X. Misinformation was operationalized here *as information that promoted cancer treatment that was known as ineffective or information that suggested cancer causes not supported by current scientific evidence* [23]. This definition focused our investigation on misinformation that could be harmful to patients with cancer or cancer survivors. Based on this operationalization, we searched existing resources that summarized unproven cancer therapy, such as “List of unproven cancer therapy” [24], a list of “Illegally sold cancer drugs” [25], and previous literature [23,26]. We extracted keywords and constructed 176 queries associated with unproven cancer treatments (Multimedia Appendix 2). Using these queries, we randomly selected up to 500 posts per query from social media. We used R software (R Foundation for Statistical Computing) to access the Academic X API. The data were manually evaluated to determine their relevance to the cancer context and unproven therapies. Queries were edited to ensure relevance. Upon corrections, the data collection was implemented on a schedule every other week between July 2022 and August 2023. After data collection was completed, the duplicate posts were removed.

#### Data Labeling

To distinguish posts with misinformation from other discussions, 2 reviewers (IF and CR) double-coded a randomly chosen subset of 1064 posts, achieving an acceptable interrater agreement of 0.68 measured with Krippendorff a [27]. Since the agreement was rather on a lower bound, we followed the current recommendations [28] and resolved disagreements between coders during research meetings, reaching consensus case by case. The initial criterion for coding misinformation was developed deductively based on the definition of misinformation used in this study. A post was coded as containing misinformation if it promoted an unproven therapy as a cancer-directed treatment. For example, a post claiming that an alkaline diet can eliminate cancer would be classified as misinformation: “Cure for cancer is an alkaline diet and lots of alkaline water.” As reviewers worked with the data, they developed additional criteria based on observed patterns. Specifically, posts were labeled as containing misinformation if they discussed unproven approaches to prevent cancer, for example, “Pygeum Bark is nature’s defense against prostate cancer.” Furthermore, if a post contained a combination of factual and false information it was labeled as “misinformation.”

Posts that were labeled as non-misinformation fell into 4 distinct categories. First, posts mentioned complementary and alternative medicine but did not promote it as a cancer treatment, for example, “Acupuncture and acupressure seem to be helpful in reducing pain and anxiety in patients having surgery.” Second, posts that used sarcasm and actively debunked misinformation related to cancer were in the non-misinformation category, for example, “If what you stated is true, then Gerson treatment for cancer is false.” The third category included posts that discussed complementary and alternative therapies but not in the context of promotion of cancer treatment, for instance, “Grapes can help protect you from the sun! Who knew?” Finally, posts that presented information with ambiguity, lack of clarity, or insufficient context were categorized as non-misinformation, for instance, “As a pancreatic cancer patient providing myself with all the additional holistic care practices made all the difference.” The author did not specify whether his symptoms were alleviated or cancer progression was slowed down because of holistic practices. Therefore, the post was coded as non-misinformation.

Once a subset of the database was labeled by 2 reviewers (IF and RC), we applied an algorithm to populate labels to the entire database. We worked with BERT [29], a machine learning model for natural language processing. The BERT model was chosen because it (1) worked well with short, informal text [30]; (2) was shown to be applicable to medical text extracted from X [31]; and (3) was successfully used in previous research to identify misinformation on X [32]. The BERT model was implemented with the programming language Python (Python Software Foundation). The manually prelabeled subset served as training data for the BERT model. Such semisupervised approaches are commonly used in similar classification tasks [33]. After training, BERT used its understanding of the language and context learned from the large corpus it was originally trained on and the specific examples from the manually prelabeled dataset. BERT predicted labels for each post in the rest of the data (unlabeled dataset), determining whether each was likely to contain misinformation or not based on the patterns and features it learned from the manually coded dataset.

After BERT algorithm assigned labels to the posts, a researcher (IF), blinded to the model’s results, manually coded a random subset of the posts (n=960) using the same “misinformation” and “non-misinformation” labels, adhering to the same criteria that were used to prelabel the data. When compared with manual coding, the algorithm identified misinformation with an accuracy of 83%, with a higher 86% specificity, and a slightly lower sensitivity of 82%. Upon labeling, 2 datasets were created and used in the first experiment: the misinformation dataset included only posts with misinformation, and control BERT dataset 1 included only posts with non-misinformation (Figure 1).

#### Data Collection: Posts From Cancer Centers

Following the definition of misinformation as “information not supported by scientific evidence or expert consensus” [34] and the definition used for this research, we assumed that posts originating from cancer centers reflect scientific evidence and expert consensus. To collect posts with factual information, we retrieved X data posted by cancer centers. Cancer centers often shared internal announcements and organizational news on X. To make posts comparable between the dataset with misinformation and control datasets, we used the keywords “cancer,” “treatment,” “chemotherapy,” “healing,” and other words related to treating cancer or controlling cancer progress. With the help of R software, we sampled 300 posts per cancer center between June 2011 and November 2022. A researcher (IF) manually checked randomly chosen (n=100) posts. As expected, no misinformation was found in the posts originating from cancer centers. The dataset, therefore, was assumed to consist of non-misinformation posts from cancer centers and was designated as control dataset 2, which was used in the second experiment alongside the misinformation dataset.

### Linguistic Characteristics Modeling

Upon data collection and labeling, we used algorithmic approaches to model linguistic characteristics. First, we used an automated text search using regular expressions in Python [35] to capture digital numbers, hashtags, and URLs in the text.

Second, we used the Linguistic Inquiry and Word Count (LIWC) software [36]. LIWC calculates the proportion of the words in the posts associated with distinct psychological dimensions [37]. In this study, LIWC identified when authors of posts used certain, absolute, or tentative language.

Third, we leveraged the natural language processing tool, Name Entity Recognition [38], which was trained on human-labeled datasets to extract names from unstructured text. Using Name Entity Recognition, we were able to identify which posts contained personal names, organizational names, or locations identified from text.

Fourth, we experimented with several models for sentiment analysis and identified the DistilBERT algorithm as an optimal approach for its accuracy in handling health-related X data [39]. DistilBERT is a black-box algorithm that is trained on a large corpus of data and is based on multiple deep stack layers. The DistilBERT algorithm identified positive, negative, and neutral tones present in the posts. To evaluate the algorithm’s performance, we manually labeled 300 posts across the databases. On average, the DistilBERT algorithm achieved an 83% accuracy (82% for misinformation and 84% for the control database) in detecting the emotional tone within the posts.

### Linguistic Characteristics Testing: Prediction of Misinformation Labels

Identified linguistic characteristics were used in an algorithm to test whether these could distinguish misinformation in posts. As shown in Figure 1, we conducted 2 experiments using *tidymodels* package in R software [40]. Using linguistic characteristics as predictors, we forecast the “misinformation” and “non-misinformation” labels in the datasets semimanually coded by researchers and BERT classification algorithm. Data were split 60:40 to enable evaluation of the predictive power of the model and reported the accuracy as a ratio of correctly classified posts to the total number of posts. We also reported area under the curve (AUC), which accounted for both false-positive and false-negative predictions. AUC value ranged from 0 to 1, where 0.5 indicated that the model performs no better than a random chance, and 1 was a perfect prediction. The model was built on the basis of Lasso (“Least Absolute Shrinkage and Selection Operator”) regression, which allowed variable selection by shrinking the coefficients of less important predictors to zero [41]. Bootstrapping procedure was applied to optimize and stabilize the selection of variables [42]. Lasso was chosen to address multicollinearity and overfitting issues in the regression model. More importantly, Lasso regression helped identify a set of linguistic characteristics that effectively distinguished posts containing misinformation. To evaluate the significance of specific linguistic characteristics, we computed importance scores, with higher scores indicating greater relevance in distinguishing posts containing misinformation. Importance scores, a common measure in predictive modeling, indicates to what extent individual predictors contribute to the overall model performance. The assessment involves permutating the characteristic values through shuffling and measuring the subsequent decline in model performance, effectively revealing the critical factors influencing predictions. Finally, we conducted a permutation statistical test (with 1000 permutations) to determine whether models with linguistic characteristics significantly outperformed random chance.

## Results

### Structured Literature Review

A total of 5677 citations were initially identified across all databases. After removing 1598 duplicates, we screened 4070 unique citations in Covidence. Subsequently, 3605 were excluded during the title and abstract review phase, leaving 464 papers for full-text review. Ultimately, we extracted linguistic characteristics from 88 full-text papers. These papers featured algorithmic approaches for identifying misinformation through automated text analysis, spanning various contexts, including politics, social issues, and computer science. Exclusion reasons are detailed in Figure 2, and additional information about the included papers can be found in Multimedia Appendix 3.

### Identified Linguistic Characteristics

The extracted linguistic characteristics and corresponding literature are detailed in Table 1. Representative examples that contain each linguistic characteristic were chosen by selecting posts from the misinformation dataset. We used results from linguistic characteristic modeling to identify such posts. The first category of characteristics pertains to the sentiment and emotional expression in the text and includes positive, negative emotions, and neutral sentiments (absence of either). Some papers delved into more nuanced emotions such as anger, fear, surprise, and others. We excluded these emotions due to the potential difficulty for readers to detect nuanced emotions reliably in the text.

The next category comprises linguistic characteristics that pertain to psychological concepts. It is worth noting that some psychological concepts consist of a combination of linguistic characteristics, such as social processes including references to family, friends, other people, and verbs indicating interactions. Although algorithms frequently use such combinations, we decided to exclude the following psychological concepts that consisted of combinations of linguistic characteristics such as cognitive, perceptual, social processes, and morality or deception. The rationale behind this exclusion is that users are unlikely able to observe and combine linguistic characteristics for evaluations of the posts. We also excluded characteristics mentioned in fewer than 4 studies, such as gratitude, insight, causation, and persuasion. Following our 3 criteria, we included negations, tentativeness, profanity (as a proxy of informality), and words associated with absolutes and certainty.

Other categories that met our inclusion criteria were linguistic characteristics such as names of individuals, locations, and organizations, as well as categories related to the presence of URLs, hashtags, personal pronouns, and numbers. Readers can identify these characteristics without additional efforts (observability criterion) and use them for evaluation of the text (applicability) because the presence of these characteristics in social media has historically been a distinguishable factor in detecting misinformation. Furthermore, these characteristics were not context-dependent and, therefore, satisfy the generalizability criterion.

**Table 1:** Linguistic characteristics and examples of misinformation.

Characteristics			Examples of linguistic characteristics and posts with misinformation^a^		Studies using characteristics for misinformation detection
**Sentiment^b^**					[43-93]
	Negative emotions	Chemo is costly and very painful. It seems to worsen illness and hasten life’s end. Sad this happened, to overcome cancer, consider utilizing cannabis oil in combination with vitamin B17. Feeling frustrated that insurance doesn’t cover certain treatments I believe in. Wish there were more options beyond the conventional cut, burn, and poison approach.			
	Positive emotions	Cure for cancer that works holistically, Vitamin B17, very good! Please do some heavy doses of medical organic marijuana if possible let it eat that cancer. Wishing you healing and joy and comfort. Wonderful treatment! Discover the incredible benefits of ProstateRelax, a natural herbal treatment for prostate cancer. ProstateRelax effectively treats and prevents the progression of prostate cancer.			
	Neutral emotions	Anyone with cancer. Check your body’s pH level. Drink alkaline water, eat alkaline foods, and avoid acidic sugary treats and dairy. Cancer cells thrive in low oxygen environments. B17, found in apricot seeds, can help. Antineoplastons, a protein suppressed by cancer, could hold the key to a potential cure.			
**Psycholinguistic**					
	Negation	Unlock the potential of Acupuncture to modulate immunity and create an environment where cancer cannot thrive. Discover the holistic power of this ancient practice in bolstering your body's defenses against cancer. I wonder why aren’t we utilizing hyperbaric chambers for Cancer? Ask your doctor about the incredible potential of pure oxygen in rejuvenating and generating new cells to combat this disease. Don’t consume sugar (as cancer thrives on it), minimize or eliminate carb-rich foods like bread and pasta, and limit alcohol intake. Embrace the power of fasting to allow your body to heal itself.		[46,49,53,70,79,81,94-96]	
	Tentativeness	3 women with similar cancer, undergoing comparable treatments—2 passed away, but 1 is thriving Possible factor? She incorporated mistletoe & other non-pharma medicines into her regimen. Concerns about [standard treatment] as a cancer solution persist, with claims of it being a harmful creation backed by influential medical forces. If it truly worked, wouldn’t it have been banned long ago like Laetrile? Listen or not: Vitamin B17, found in Apricot seeds and sold online as a “health supplement,” has caught my attention as a potential cancer cure.		[49,51,59,61,62,66,81,94,96-100]	
	Absolute language or certainty	I take sea buckthorn pills! They are an absolute lifesaver. Vitamin B17 has definitely prevented my cancer from spreading. It's been a while, and there has been no growth. During my time in a chemo clinic, alternative treatments were never allowed to be discussed or promoted. I left and started studying herbal medicine.		[43,51,59,61,94,97-101]	
	Profanity	Create an alkaline environment that cancer can’t thrive in! Incorporate herbs, vitamins, and minerals to support your healing journey. You are going to heal and beat that s*** Go to a poor country and you get real tea with real ginger. Go to a rich country and you will get chemical b**** that will give you cancer It damages healthy cells, no surefire cancer cure. It's like a c*** shoot for survival & recurrence. But I choose a different path: starving cancerous cells with therapeutic fasting & lifestyle shifts.		[48,57,62,63,66,69,81,89,96,98,102]	
**Named entities**					[44,49,51,60,64,69,79,93,103-109]
	Names	I watched the documentary of Dr. B [name] on YouTube. He cured stage 4 cancer with no chemotherapy and no radiation.			
	Location	Fascinating, study from M [name of State]! Certain sound frequencies may aid the body in fighting cancer. Pair this with an alkaline diet - and the world is cured!			
	Organization	Must-watch documentary on YouTube! Unveiling a shocking cancer cure cover-up for over 40 years! B [name]: The Cancer Cure Cover-Up—Full documentary available now!			
**URL**			Insights from Dr. N [name]! Learn how to transform the cancer terrain, boost immunity, and create an inhospitable environment for cancer using Acupuncture, Chinese herbal medicines, and food therapies. Check out the discussion here: [link provided].		[45,51,52,54,55,62,69,78,79 ,86-88,92,93,98,99,101,104,107-117]
**Numeric data**			Cancer is nearly 100% curable but beware of certain hospital treatments. Explore alternative options for better outcomes.		[44,49,51,57,65,67,70,72,73,79,81,94,98,101,105]
**Pronouns**			I love your positivity and your fight against cancer. Keep up the fight and adhere to Alkaline Diet for a healthier journey. Your cancer can be cured by #fasting paired with no sugar alkaline diet. A pro basketball player revealed how organic Wheatgrass healed his close friend from blood cancer. A testament to the power of natural remedies!		[61,66,68,72,78,79,93,97,99,103,106,108,112,118-121]
**Hashtag**			#TualangHoney helps against skin Cancer with no side effects.		[43,44,47,52-55,59,64,66,77-79,82,87, 92,96,98,101,104,107,108,111,115,119,122,123]

^a^All posts were paraphrased to protect the author’s anonymity.

^b^In sentiment analysis, emotions are identified by a “black box” model (DistilBERT). While we report here examples and highlight “negative/positive” words in the sentence, we must acknowledge that the algorithm may or may not use these words for detecting emotions.

### Collected Data From X

We collected a total of 45,791 posts related to unproven cancer therapies. Among these, 13,046 posts were labeled as misinformation (forming the misinformation dataset), while 32,745 posts were categorized as non-misinformation (comprising control dataset 1). Furthermore, we gathered 6782 posts from the profiles of comprehensive cancer centers, which were used as control dataset 2, as shown in Figure 1. The content description of both the misinformation dataset and the control dataset 1 is shown in Table 2. To illustrate the dataset in this study, we categorized the X posts into 9 distinct categories. The examples of the posts with misinformation are shown in Table 1.

**Table 2:** Relevant prevalence of therapy categories within posts about unproven cancer therapy.

Categories of therapies	Total posts, n	Posts with misinformation, n (%)^a^	Examples of unproven cancer therapy
Diet based	5179	3069 (59)	Antioxidant, fasting, and alkaline diet
Alternative health system	7036	2250 (32)	Herbal therapy and ayurveda
Plant- and fungus-based	13,851	4386 (32)	Mushrooms
Synthetic substances	8471	2637 (31)	Antineoplastic Brudzinski and vitamin C
Spiritual and mental healing	2347	272 (12)	Meditation, praying, and tai chi
Electromagnetic and energy-based	2825	283 (10)	Polarity therapy and magnetic
Physical procedures	1144	49 (4)	Acupuncture
Other	4938	100 (2)	N/A^b^
Total	45,791	13,046 (28)	N/A

^a^Out of the total number of posts.

^b^N/A: not applicable.

### Linguistic Characteristics Testing: Prediction of Misinformation Labels

As shown in Table 3, experiment 1 demonstrated that linguistic characteristics predicted misinformation with 60% accuracy. In experiment 2, they exhibited even stronger predictive power, achieving an accuracy of 77%. The importance scores for each linguistic characteristic are shown in Table 4.

Next, we selected linguistic characteristics with an impact score 0.05 and consistent predictive performance across experiments 1 and 2. These short-listed characteristics underwent further testing within the same experiments. In experiment 1, the short-listed characteristics achieved an accuracy rate of 50%, which did not significantly differ from random chance (*P*>.90). However, in experiment 2, these characteristics predicted misinformation with an accuracy rate of 73% and an AUC of 83. This performance was significantly better than random chance (McNemar ^2^_1_=5.7 ×10^7^; *P*<.001). The importance scores for the short-listed characteristics are shown in Table 4. For a more detailed breakdown of the importance scores, we have summarized the percentage of posts containing these short-listed characteristics by dataset in Table 4 and the complete list in Multimedia Appendix 4.

**Table 3:** Lasso regression performance.

Name of the dataset	Total posts, n	Posts with misinformation, n	Accuracy, %
Experiment 1: misinformation dataset and control dataset 1	45,791	13,046	60
Experiment 2: misinformation dataset and control dataset 2	19,828	13,046	77

**Table 4:** Importance scores.

Linguistic characteristics	Experiment with control group 1		Experiment with control group 2		Experiment with short-listed characteristics (control group 2)	
	Predictors		Predictors		Predictors	
	Negative	Positive	Negative	Positive	Negative	Positive
Absolute language	—^a^	*0.11* ^b^	—	*0.69*	—	*0.84*
Certainty	—	*0.21*	—	*1.13*	—	*1.02*
First-person pronoun	0.27	—	—	1.31	—	—
Hashtags	*0.56*	—	*1.55*	—	*1.6*	—
Location	*0.27*	—	*0.27*	—	*0.46*	—
Name	—	0.08	0.91	—	—	—
Negation	0.53	—	—	0.73	—	—
Negative emotions	0.24	—	0	—	—	—
Neutral emotions	0	—	—	0.07	—	—
Number	—	*0.17*	—	*0.29*	—	*0.28*
Organization	—	0.02	0.63	—	—	—
Positive emotions	—	0.31	0.46	—	—	—
Profanity	0.92	—	—	1.99	—	—
Second-person pronoun	0.02	—	0.45	—	—	—
Tentativeness	*0.08*	—	*0.16*	—	*0.08*	—
Third-person pronoun	0	—	0.23	—	—	—
URL	*0.3*	—	*2.28*	—	*2.47*	—

^a^Not applicable.

^b^Italicized values represent short-listed characteristics.

**Table 5:** The percentage of posts with short-listed linguistic characteristics.

Linguistic characteristics			Misinformation dataset (n=13,046), n (%)		Control dataset 1 (n=32,745), n (%)		Control dataset 2 (n=6782), n (%)
**Positive predictors**							
	Certainty	1579 (12)		3044 (9)		208 (3)	
	Absolute	2741 (21)		7294 (22)^a^		630 (9)	
	Number	6358 (49)		14,360 (44)		2497 (37)	
**Negative predictors**							
	URL	6978 (53)		19,591 (60)		6560 (97)	
	Hashtags	2296 (18)		8512 (26)		4343 (64)	
	Location	1212 (9)		3373 (12)		975 (14)	
	Tentativeness	4154 (32)		11,171 (34)		1835 (27)^a^	

^a^Valence of predictions is inferred from the model, which includes all characteristics simultaneously.

## Discussion

### Principal Findings

We have identified linguistic characteristics that can help people affected by cancer detect cancer misinformation on social media platforms such as X. Linguistic characteristics that were *likely* to be present in posts with misinformation were related to certain, absolute language, and numbers. Certain language included phrases that reflected a “degree of bravado” or “boasting of certainty.” Examples of certain languages could be “I really believe,” “it is definitely helpful,” and similar others [36]. The absolute language referred to phrases that reflect black-and-white thinking and included words such as “none,” “all,” “never,” and others [36]. The number category encompassed any information reported with digits such as percentages, count of any units, years, and priorities. Notably, all 3 linguistic characteristics could be united under the umbrella of definite, confident language. Linguistic characteristics that were *unlikely* to be present in posts with misinformation encompassed URLs, hashtags, and location mentions. Each of these attributes could be considered as a form of citation or reference. URLs offered direct links to the original source or further information, hashtags connected posts to broader relevant discussions, while locations mentioned in posts provided context and a sense of origin to the information shared. Our findings are consistent with some of the suggestions provided by previous guidelines for identifying misinformation. For instance, the Food and Drug Administration recommends being vigilant if patients read confident statements such as a drug definitely “cures cancer” or “guarantees results” [124]. Other guidelines encouraged users to search for references and original sources of health-related information [12-14].

While consistent with previous recommendations, our findings make a unique contribution. Previous work has based the guidelines on theoretical assumptions, while our study is one of the first to provide some empirical evidence based on a large dataset to support the recommendations for users. Another contribution is that we outlined ineffective linguistic characteristics for detecting cancer misinformation. Despite a substantial body of research showing that social media posts with sentiments predicted fake news, we did not find these relationships. A potential explanation could be the algorithm’s limited efficiency in identifying emotions within cancer-related contexts. Furthermore, it is possible that authors express a limited range of emotions in cancer-related conversations, typically negative emotions toward cancer and both positive and negative emotions toward various treatments, including those that are unproven. These emotions may vary little across posts containing valid and nonvalid information, making emotions an unreliable factor for distinguishing misinformation.

Our work accumulates knowledge about misinformation detection from the literature covering a wide range of contexts—including political, social, and computer science—and translates this knowledge to the cancer context. The findings highlighted promising avenues for future research and could expedite the development of automated and augmented methods for identifying and verifying cancer-related misinformation on social media platforms. Finally, the robust labeled datasets developed by our research team are available to other researchers upon request to the corresponding author, thereby further supporting research on misinformation within the context of cancer and social media.

In practice, our work is at the forefront of customizing recommendations and contextualizing them for social network users. Our exploratory findings suggest a promising direction for studying linguistic characteristics that information users might apply when making quick judgments while scrolling through X feeds. Empowering users to stay vigilant in their initial evaluations could help reduce the spread of misinformation and the formation of erroneous beliefs. This is a crucial area for future research, which should explore how these findings apply in different cancer-related contexts and across various social networks.

### Limitations

All the studies included in our analysis exclusively originate from peer-reviewed journals and conference proceedings; however, we must exercise caution when considering the potential for publication bias. Furthermore, in accordance with our selection criteria for linguistic characteristics, we included only those papers that focused on text and excluded other forms of social media content, such as videos and images. We recommend that future research comprehensively explore social media, including multimedia content, as it could potentially provide additional insights for user-friendly recommendations.

In selecting linguistic characteristics, we prioritized observability, applicability, and generalizability. However, alternative criteria may be considered when users are open to a more thorough exploration of a post’s validity. For example, future research should explore the use of metadata, link content analysis, and hashtag meanings. As misinformation evolves and its authors adjust to societal changes, the linguistic characteristics that identify misinformation may also shift. A longitudinal analysis is necessary to understand how linguistic characteristics perform in predicting misinformation over time.

Algorithms used in our analysis operate with a certain level of accuracy. Specifically, the accuracy of label identification in the dataset reached 83%, indicating that approximately 17% of posts were labeled incorrectly. This means that in experiment 1 some proportion of misinformation is included in the non-misinformation group and vice versa, making further exploration less accurate in experiment 1. This degree of uncertainty is common in algorithmic performance. Therefore, it is important to interpret our results in light of the inherent imperfections in algorithmic performance.

Furthermore, we encountered that the short-listed linguistic characteristics did not significantly outperform random chance in identifying misinformation in experiment 1. This outcome underscores a potential boundary condition of the effectiveness of the linguistic characteristics. Notably, experiment 1 encompassed more homogeneous data in contrast to experiment 2. Based on these findings, it becomes plausible to speculate that linguistic characteristics might provide limited help when a reader assesses posts within a closely knit community.

In experiment 2, the control dataset 2 consisted of posts shared by cancer centers and was compared with the misinformation dataset comprising random posts. To address this limitation, we collected posts from cancer centers that contain words related to cancer therapies. This step was taken to ensure a similar context of discussion as the posts with unproven therapy. Next, we exclude linguistic characteristics that are likely displayed differences between datasets due to the distinct nature of the information within control dataset 2. For example, linguistic traits such as “the use of profanity” or “first-person pronouns” were discarded. Furthermore, we decided to focus our analysis solely on the text within the posts and omitted other accompanying metainformation that users might observe, such as the user’s name, location of the author, and posting time. This approach allowed us to assume that posts shared by cancer centers might be perceived more broadly, for instance, as posts shared by researchers, physicians, administrators, and patient advocates. Because of these measures, we anticipate that the linguistic characteristics identified in this research may help differentiate between health misinformation and factual posts on social media, irrespective of their sources. Despite our precautionary measures, we cannot fully guarantee that identified linguistics characteristics certainly distinguish between posts with misinformation and non-misinformation versus posts produced by the general public and posts by health experts from health care systems. However, there are factors that support the first conclusion more than the second. First, our findings are consistent with the previous theoretical and practical recommendations for identifying misinformation [12-14]. Second, the associated with misinformation linguistic characteristics, such as numbers and assertive language, are expected to be used by health experts. For instance, providers use numbers more confidently than the general public [125]. Professional guidelines for health providers encourage them to use numbers over verbal descriptions [126] as well as the use of assertive language in communication with patients [127,128]. Yet, our study associated these characteristics with misinformation shared by the general public on social media, which suggests that we might be finding more than just a mere distinction between the general public language and the health professional language. One study in and of itself is not yet a comprehensive body of evidence. Our findings will need to be validated and built upon via additional studies—including those that use posts from other types of entities and comparison groups.

Finally, our data were collected only on a single social network X. Many characteristics and customs of X are transferable to other social networks and our recommendations are likely to go beyond application on X, as demonstrated by the consistency of our recommendations with the recommendations of other researchers [12-14]. Given this limitation, our results need to be generalized cautiously, and further similar research is needed for different platforms (eg, Facebook, Pinterest, etc).

### Conclusions

Our structured review synthesized knowledge from studies that used algorithmic approaches for text analysis to detect misinformation in social media. From this literature, we identified user-friendly linguistic characteristics that can assist individuals in distinguishing misinformation when they seek health-related information on social media. The linguistic characteristics, such as certainty, absolute language, and numbers, were positively associated with misinformation, while characteristics such as URLs, hashtags, and location mentions were negatively predictive of misinformation. Based on these findings, we suggested that users should be cautious of social media posts containing confident promises or specific numbers without proper references to the original information. According to our analysis, we expect that this approach will allow users to filter out two-thirds of posts with cancer-related misinformation. Yet, before drawing a definitive conclusion, further testing with different datasets is required.

## Appendix

Multimedia Appendix 1 Strategy for literature review.

Multimedia Appendix 2 List of unproven therapy.

Multimedia Appendix 3 Summary of the literature.

Multimedia Appendix 4 Summary of linguistic characteristics.

## Abbreviations

API: application programming interface
AUC: area under the curve
BERT: Bidirectional Encoder Representations from Transformers
LIWC: Linguistic Inquiry and Word Count
PRISMA: Preferred Reporting Items for Systematic Reviews and Meta-Analyses
