# Supplementary material for: Identifying Misinformation About Unproven Cancer Treatments on Social Media Using User-Friendly Linguistic Characteristics: Content Analysis
Source: JMIR Infodemiology. 2025 Feb 12;5:e62703. doi: 10.2196/62703 (PMC11888050; doi:10.2196/62703)
Supplement: Multimedia Appendix 1 [file infodemiology_v5i1e62703_app1.docx]

# Appendix 1: Structured Literature Review

### **Keywords**

| Concept | # | Keywords | Search in |
| --- | --- | --- | --- |
| Text analyzed | 1 | “Digital media” OR Facebook OR Journalism OR Magazine OR News OR Newspaper OR Reddit OR Tabloid OR Tabloids OR Telegram OR Tumblr OR Twitter OR Tweet OR Tweets | Title/abstract/ keywords |
| Misinformation | 2 | Bias OR Biased OR Biases OR Bogus OR Censor OR Censors OR Censorship OR “Click bait” OR “Click baits” OR “click bites” OR “click bytes” OR clickbait OR clickbaits OR conspiracies OR conspiracy OR Credibility OR Credible OR Deceive OR Deception OR Deceptive OR disinformation OR Fabricated OR Fabrication OR “Fact check” OR “Fact checked” OR “Fact checking” OR Fake OR “fake news” OR Fallacies OR Fallacy OR False OR “false information” OR Forgeries OR forgery OR Fraudulent OR Hoax OR Imposter OR junk OR lying OR malinformation OR Manipulated OR Manipulated OR Manipulation OR “Manufactured amplification” OR misinformation OR mislead OR misleading OR misreporting OR Phony OR propaganda OR Pseudoscience OR rumor OR Rumour OR Unverified OR Verification OR Verified OR Verify | Title/abstract/ keywords |
| Algorithm/ linguistic features | 3 | Algorithm OR Algorithms OR “automated deception detection” OR “Automated text analysis” OR automation OR “Bidirectional Encoder Representations from Transformers” OR “classification algorithm” OR “deep learning” OR “feature extraction” OR “feature modeling” OR “k-Nearest Neighbor” OR “LightGBM” OR “logistic regression” OR “Machine learning” OR “natural language” OR “natural language processing” OR “Natural language processing systems” OR “natural text” OR “natural text processing” OR “neural network” OR Neural networks OR “NLP” OR “random forest” OR “sentiment analysis “ OR “support vector machine” OR “support vector machines” OR “support vector network” OR “support vector networks” OR “support-vector machine” OR “support-vector machines” OR “support-vector network” OR “support-vector networks” OR “text modeling” | Title/abstract/ keywords/ metadata |
| Internet | 4 | internet OR online OR Platform OR “Social media” OR “Social network” OR “social networks” OR web | Title/abstract/ keywords/ metadata |
| Features | 5 | Dictionary OR Feature OR Features OR “Linguistic Inquiry and Word Count” OR LIWC OR “Named Entity Recognition” OR Semantic OR Sentiment OR “textual properties” OR “textual property” OR “Topic model” OR “Topic modeling” OR “topic modelling” OR “Word sketch” OR “word sketches” OR “word sketching” | Title/abstract/ keywords/ metadata |
|  | 6 | 1 AND 2 AND 3 AND 4 AND 5 |  |
| Date | 7 | 2012-2022 |  |
|  |  | 6 AND 7 |  |

### **Search Strategy**

The search strategies will be created and implemented by a librarian with expertise in systematic review searching. We include here criteria and keywords that are planned to be used**.** Before implementing the strategy.

**Include:** Original research articles published in scientific journals or at conferences

**Exclude:** Workshops; poster presentations; blogs; magazines, review articles, books, book chapters

**Year:** 2012-2022

**Language:** English

**Search**: in title, abstract, and keywords

**Databases:**

1. ProQuest Central (ProQuest)
2. Scopus (Elsevier)
3. IEEE Xplore (Institute of Electrical and Electronics Engineers)
4. ACM Digital Library Guide to Computing Literature (Association for Computing Machinery)
5. Communication and Mass Media Complete (EBSCOhost)

| **Database** | **Results before deduplication** | **Results after deduplication** |
| --- | --- | --- |
| ProQuest Central | 1510 | 1445 |
| Scopus | 1470 | 868 |
| IEEE Xplore | 1086 | 497 |
| ACM Digital Library | 1602 | 1251 |
| Communication & Mass Media Complete | 22 | 9 |
| Total | 5,677 | 4,070 |

### ProQuest Central

Search conducted December 2, 2022

| # | Search terms | # of results |
| --- | --- | --- |
| 1 | TI("Digital media” OR Facebook OR Journalism OR Magazine OR News OR Newspaper OR Reddit OR Tabloid OR Tabloids OR Telegram OR Tumblr OR Twitter OR Tweet OR Tweets) | 20,715,503 |
| 2 | AB("Digital media” OR Facebook OR Journalism OR Magazine OR News OR Newspaper OR Reddit OR Tabloid OR Tabloids OR Telegram OR Tumblr OR Twitter OR Tweet OR Tweets) | 846,608 |
| 3 | SUBJECT(“Digital media” OR news) | 13,433,084 |
| 4 | TI(Bias OR Biased OR Biases OR Bogus OR Censor OR Censors OR Censorship OR “Click bait” OR “Click baits” OR “click bites” OR “click bytes” OR clickbait OR clickbaits OR conspiracies OR conspiracy OR Credibility OR Credible OR Deceive OR Deception OR Deceptive OR disinformation OR Fabricated OR Fabrication OR “Fact check” OR “Fact checked” OR “Fact checking” OR Fake OR “fake news” OR Fallacies OR Fallacy OR False OR “false information” OR Forgeries OR forgery OR Fraudulent OR Hoax OR Imposter OR junk OR lie OR lying OR malinformation OR Manipulated OR Manipulated OR Manipulation OR “Manufactured amplification” OR misinformation OR mislead OR misleading OR misreporting OR Phony OR propaganda OR Pseudoscience OR rumor OR Rumour OR Unverified OR Verification OR Verified OR Verify) | 2,617,859 |
| 5 | AB(Bias OR Biased OR Biases OR Bogus OR Censor OR Censors OR Censorship OR “Click bait” OR “Click baits” OR “click bites” OR “click bytes” OR clickbait OR clickbaits OR conspiracies OR conspiracy OR Credibility OR Credible OR Deceive OR Deception OR Deceptive OR disinformation OR Fabricated OR Fabrication OR “Fact check” OR “Fact checked” OR “Fact checking” OR Fake OR “fake news” OR Fallacies OR Fallacy OR False OR “false information” OR Forgeries OR forgery OR Fraudulent OR Hoax OR Imposter OR junk OR lying OR malinformation OR Manipulated OR Manipulated OR Manipulation OR “Manufactured amplification” OR misinformation OR mislead OR misleading OR misreporting OR Phony OR propaganda OR Pseudoscience OR rumor OR Rumour OR Unverified OR Verification OR Verified OR Verify) | 1,624,418 |
| 6 | SUBJECT(disinformation OR “fake news” OR misinformation) | 17,757 |
| 7 | (1 AND 4) OR (2 AND 5) OR (3 AND (4 OR 5 OR 6)) OR ((1 OR 2 OR 3) AND 6) | 223,120 |
| 8 | NOFT(Algorithm OR Algorithms OR “automated deception detection” OR “Automated text analysis” OR automation OR “Bidirectional Encoder Representations from Transformers” OR “classification algorithm” OR “deep learning” OR “feature extraction” OR “feature modeling” OR “k-Nearest Neighbor” OR “LightGBM” OR “logistic regression” OR “Machine learning” OR “natural language” OR “natural language processing” OR “Natural language processing systems” OR “natural text” OR “natural text processing” OR “neural network” OR Neural networks OR “NLP” OR “random forest” OR “sentiment analysis “ OR “support vector machine” OR “support vector machines” OR “support vector network” OR “support vector networks” OR “support-vector machine” OR “support-vector machines” OR “support-vector network” OR “support-vector networks” OR “text modeling”) | 4,606,347 |
| 9 | NOFT(internet OR online OR “Social media” OR “Social network” OR “social networks” OR web) | 36,347,795 |
| 10 | NOFT(Dictionary OR Feature OR Features OR “Linguistic Inquiry and Word Count” OR LIWC OR “Named Entity Recognition” OR Semantic OR Sentiment OR “textual properties” OR “textual property” OR “Topic model” OR “Topic modeling” OR “topic modelling” OR “Word sketch” OR “word sketches” OR “word sketching”) | 15,979,579 |
| 11 | 7 AND 8 AND 9 AND 10 | 1,626 |
|  | Limited by: Source type: Books, Conference Papers & Proceedings, Scholarly Journals, Working Papers | 1,520 |
|  | Limited by: Date: After 2012 | 1,510 |

### Scopus

Search conducted December 2, 2022

| # | Keywords | Number of results |
| --- | --- | --- |
| 1 | TITLE-ABS-KEY (“Digital media” OR Facebook OR Journalism OR Magazine OR News OR Newspaper OR Reddit OR Tabloid OR Tabloids OR Telegram OR Tumblr OR Twitter OR Tweet OR Tweets) | 333,859 |
| 2 | TITLE-ABS-KEY (Bias OR Biased OR Biases OR Bogus OR Censor OR Censors OR Censorship OR “Click bait” OR “Click baits” OR “click bites” OR “click bytes” OR clickbait OR clickbaits OR conspiracies OR conspiracy OR Credibility OR Credible OR Deceive OR Deception OR Deceptive OR disinformation OR Fabricated OR Fabrication OR “Fact check” OR “Fact checked” OR “Fact checking” OR Fake OR “fake news” OR Fallacies OR Fallacy OR False OR “false information” OR Forgeries OR forgery OR Fraudulent OR Hoax OR Imposter OR junk OR lying OR malinformation OR Manipulated OR Manipulated OR Manipulation OR “Manufactured amplification” OR misinformation OR mislead OR misleading OR misreporting OR Phony OR propaganda OR Pseudoscience OR rumor OR Rumour OR Unverified OR Verification OR Verified OR Verify) | 4,251,315 |
| 3 | TITLE-ABS-KEY (Algorithm OR Algorithms OR “automated deception detection” OR “Automated text analysis” OR automation OR “Bidirectional Encoder Representations from Transformers” OR “classification algorithm” OR “deep learning” OR “feature extraction” OR “feature modeling” OR “k-Nearest Neighbor” OR “LightGBM” OR “logistic regression” OR “Machine learning” OR “natural language” OR “natural language processing” OR “Natural language processing systems” OR “natural text” OR “natural text processing” OR “neural network” OR Neural networks OR “NLP” OR “random forest” OR “sentiment analysis “ OR “support vector machine” OR “support vector machines” OR “support vector network” OR “support vector networks” OR “support-vector machine” OR “support-vector machines” OR “support-vector network” OR “support-vector networks” OR “text modeling”) | 1,613,673 |
| 4 | TITLE-ABS-KEY (internet OR online OR Platform OR “Social media” OR “Social network” OR “social networks” OR web) | 2,852,761 |
| 5 | TITLE-ABS-KEY (Dictionary OR Feature OR Features OR “Linguistic Inquiry and Word Count” OR LIWC OR “Named Entity Recognition” OR Semantic OR Sentiment OR “textual properties” OR “textual property” OR “Topic model” OR “Topic modeling” OR “topic modelling” OR “Word sketch” OR “word sketches” OR “word sketching”) | 4,529,696 |
| 6 | 1 AND 2 AND 3 AND 4 AND 5 | 1,489 |
| 7 | Filter applied: PUBYEAR AFT 2011 |  |
|  | 6 AND 7 | 1,474 |

### IEEE Xplore

Search conducted December 2, 2022

| # | Keywords | Search in |
| --- | --- | --- |
| 1 | ("All Metadata":"Digital media" OR "All Metadata":Facebook OR "All Metadata":Journalism OR "All Metadata":Magazine OR "All Metadata":News OR "All Metadata":Newspaper OR "All Metadata":Reddit OR "All Metadata":Tabloid OR "All Metadata":Tabloids OR "All Metadata":Telegram OR "All Metadata":Tumblr OR "All Metadata":Twitter OR "All Metadata":Tweet*) | 122,779 |
| 2 | (("All Metadata":Bias* OR "All Metadata":Bogus OR "All Metadata":Censor* OR "All Metadata":"Click bait" OR "All Metadata":"click bites" OR "All Metadata":"click bytes" OR "All Metadata":clickbait* OR "All Metadata":conspirac* OR "All Metadata":Credibility OR "All Metadata":Credible OR "All Metadata":Deceive OR "All Metadata":Decepti* OR "All Metadata":disinformation OR "All Metadata":Fabricated OR "All Metadata":Fabrication OR "All Metadata":"Fact check" OR "All Metadata":"Fact checked" OR "All Metadata":"Fact checking" OR "All Metadata":Fake OR "All Metadata":"fake news") OR ("All Metadata":Fallacy OR "All Metadata":Fallacies OR "All Metadata":False OR "All Metadata":forgery OR "All Metadata":Fraudulent OR "All Metadata":Hoax OR "All Metadata":Imposter OR "All Metadata":junk OR "All Metadata":lying OR "All Metadata":malinformation OR "All Metadata":Manipulat* OR "All Metadata":"Manufactured amplification" OR "All Metadata":misinformation OR "All Metadata":mislead OR "All Metadata":misleading OR "All Metadata":misreporting OR "All Metadata":Phony OR "All Metadata":propaganda OR "All Metadata":Pseudoscience OR "All Metadata":rumor OR "All Metadata":Unverified OR "All Metadata":Verify OR "All Metadata":Verification)) | 725,903 |
| 3 | ("All Metadata":Algorithm* OR "All Metadata":"automated deception detection" OR "All Metadata":"Automated text analysis" OR "All Metadata":automation OR "All Metadata":"Bidirectional Encoder Representations from Transformers" OR "All Metadata":"deep learning" OR "All Metadata":"feature extraction" OR "All Metadata":"feature modeling" OR "All Metadata":"k-Nearest Neighbor" OR "All Metadata":"LightGBM" OR "All Metadata":"logistic regression" OR "All Metadata":"Machine learning" OR "All Metadata":"natural language" OR "All Metadata":"natural text" OR "All Metadata":"neural network" OR "All Metadata":"neural networks" OR "All Metadata":NLP OR "All Metadata":"random forest" OR "All Metadata":"sentiment analysis " OR "All Metadata":"support vector" OR "All Metadata":"text modeling") | 1,712,001 |
| 4 | ("All Metadata":internet OR "All Metadata":online OR "All Metadata":"Social media" OR "All Metadata":"Social network" OR "All Metadata":"social networks" OR "All Metadata":web) | 460,893 |
| 5 | ("All Metadata":dictionary OR "All Metadata":feature OR "All Metadata":features OR "All Metadata":"linguistic inquiry and word count" OR "All Metadata":"named entity recognition" OR "All Metadata":semantic OR "All Metadata":sentiment OR "All Metadata":"textual properties" OR "All Metadata":"textual property" OR "All Metadata":"topic modeling" OR "All Metadata":"word sketch" OR "All Metadata":"word sketches" OR "All Metadata":"word sketching") |  |
| 6 | 1 AND 2 AND 3 AND 4 AND 5 | 1,120 |
| 7 | Filter applied: Date: 2012-2022 | 1,086 |

### ACM Digital Library Guide to Computing Literature

Search conducted December 2, 2022

| # | Keywords | Results |
| --- | --- | --- |
| 1 | (Title:( “Digital media” OR Facebook OR Journalism OR Magazine OR News* OR Reddit OR Tabloid* OR Telegram OR Tumblr OR Twitter OR Tweet*) OR Abstract:( “Digital media” OR Facebook OR Journalism OR Magazine OR News* OR Reddit OR Tabloid* OR Telegram OR Tumblr OR Twitter OR Tweet*)) | 27425 |
| 2 | (Title:( Bias* OR Bogus OR Censor* OR “Click bait” OR “click bites” OR “click bytes” OR clickbait* OR conspirac* OR Credibility OR Credible OR Deceive OR Decepti* OR disinformation OR Fabricated OR Fabrication OR “Fact check” OR “Fact checked” OR “Fact checking” OR Fake OR “fake news” OR Fallac* OR False OR forger* OR Fraudulent OR Hoax OR Imposter OR junk OR lying OR malinformation OR Manipulat* OR “Manufactured amplification” OR misinformation OR mislead* OR misreporting OR Phony OR propaganda OR Pseudoscience OR rumor OR Unverified OR Verif*) OR Abstract:( Bias* OR Bogus OR Censor* OR “Click bait” OR “click bites” OR “click bytes” OR clickbait* OR conspirac* OR Credibility OR Credible OR Deceive OR Decepti* OR disinformation OR Fabricated OR Fabrication OR “Fact check” OR “Fact checked” OR “Fact checking” OR Fake OR “fake news” OR Fallac* OR False OR forger* OR Fraudulent OR Hoax OR Imposter OR junk OR lying OR malinformation OR Manipulat* OR “Manufactured amplification” OR misinformation OR mislead* OR misreporting OR Phony OR propaganda OR Pseudoscience OR rumor OR Unverified OR Verif*)) | 56153 |
| 3 | (Title:( Algorithm* OR “automated deception detection” OR “Automated text analysis” OR automation OR “Bidirectional Encoder Representations from Transformers” OR “deep learning” OR “feature extraction” OR “feature modeling” OR “k-Nearest Neighbor” OR “LightGBM” OR “logistic regression” OR “Machine learning” OR “natural language” OR “natural text” OR “neural network” OR “neural networks” OR NLP OR “random forest” OR “sentiment analysis “ OR “support vector” OR “text modeling") OR Abstract:( Algorithm* OR “automated deception detection” OR “Automated text analysis” OR automation OR “Bidirectional Encoder Representations from Transformers” OR “deep learning” OR “feature extraction” OR “feature modeling” OR “k-Nearest Neighbor” OR “LightGBM” OR “logistic regression” OR “Machine learning” OR “natural language” OR “natural text” OR “neural network” OR “neural networks” OR NLP OR “random forest” OR “sentiment analysis “ OR “support vector” OR “text modeling”)) | 171962 |
| 4 | (All:(internet OR online OR Platform OR "Social media" OR "Social network" OR "social networks" OR web)) | 16644 |
| 5 | (All:( Dictionary OR Feature OR Features OR “Linguistic Inquiry and Word Count” OR LIWC OR “Named Entity Recognition” OR Semantic OR Sentiment OR “textual properties” OR “textual property” OR “Topic model” OR “Topic modeling” OR “topic modelling” OR “Word sketch” OR “word sketches” OR “word sketching”) | 399225 |
| 6 | 1 AND 2 AND 3 AND 4 AND 5 | 1,883 |
| 7 | Filter applied: 2012-2022 | 1,602 |

### Communication and Mass Media

Search conducted December 2, 2022

| # | Keywords | Search in |
| --- | --- | --- |
| 1 | TI (“Digital media” OR Facebook OR Journalism OR Magazine OR News OR Newspaper OR Reddit OR Tabloid OR Tabloids OR Telegram OR Tumblr OR Twitter OR Tweet OR Tweets ) OR AB (“Digital media” OR Facebook OR Journalism OR Magazine OR News OR Newspaper OR Reddit OR Tabloid OR Tabloids OR Telegram OR Tumblr OR Twitter OR Tweet OR Tweets ) OR SU (“Digital media” OR Facebook OR Journalism OR Magazine OR News OR Newspaper OR Reddit OR Tabloid OR Tabloids OR Telegram OR Tumblr OR Twitter OR Tweet OR Tweets ) | 155,852 |
| 2 | (TI ( Bias OR Biased OR Biases OR Bogus OR Censor OR Censors OR Censorship OR “Click bait” OR “Click baits” OR “click bites” OR “click bytes” OR clickbait OR clickbaits OR conspiracies OR conspiracy OR Credibility OR Credible OR Deceive OR Deception OR Deceptive OR disinformation OR Fabricated OR Fabrication OR “Fact check” OR “Fact checked” OR “Fact checking” OR Fake OR “fake news” OR Fallacies OR Fallacy OR False OR “false information” OR Forgeries OR forgery OR Fraudulent OR Hoax OR Imposter OR junk OR lying OR malinformation OR Manipulated OR Manipulated OR Manipulation OR “Manufactured amplification” OR misinformation OR mislead OR misleading OR misreporting OR Phony OR propaganda OR Pseudoscience OR rumor OR Rumour OR Unverified OR Verification OR Verified OR Verify ) OR AB ( Bias OR Biased OR Biases OR Bogus OR Censor OR Censors OR Censorship OR “Click bait” OR “Click baits” OR “click bites” OR “click bytes” OR clickbait OR clickbaits OR conspiracies OR conspiracy OR Credibility OR Credible OR Deceive OR Deception OR Deceptive OR disinformation OR Fabricated OR Fabrication OR “Fact check” OR “Fact checked” OR “Fact checking” OR Fake OR “fake news” OR Fallacies OR Fallacy OR False OR “false information” OR Forgeries OR forgery OR Fraudulent OR Hoax OR Imposter OR junk OR lying OR malinformation OR Manipulated OR Manipulated OR Manipulation OR “Manufactured amplification” OR misinformation OR mislead OR misleading OR misreporting OR Phony OR propaganda OR Pseudoscience OR rumor OR Rumour OR Unverified OR Verification OR Verified OR Verify ) OR SU ( Bias OR Biased OR Biases OR Bogus OR Censor OR Censors OR Censorship OR “Click bait” OR “Click baits” OR “click bites” OR “click bytes” OR clickbait OR clickbaits OR conspiracies OR conspiracy OR Credibility OR Credible OR Deceive OR Deception OR Deceptive OR disinformation OR Fabricated OR Fabrication OR “Fact check” OR “Fact checked” OR “Fact checking” OR Fake OR “fake news” OR Fallacies OR Fallacy OR False OR “false information” OR Forgeries OR forgery OR Fraudulent OR Hoax OR Imposter OR junk OR lying OR malinformation OR Manipulated OR Manipulated OR Manipulation OR “Manufactured amplification” OR misinformation OR mislead OR misleading OR misreporting OR Phony OR propaganda OR Pseudoscience OR rumor OR Rumour OR Unverified OR Verification OR Verified OR Verify )) | 39,788 |
| 3 | (TI ( Algorithm OR Algorithms OR “automated deception detection” OR “Automated text analysis” OR automation OR “Bidirectional Encoder Representations from Transformers” OR “classification algorithm” OR “deep learning” OR “feature extraction” OR “feature modeling” OR “k-Nearest Neighbor” OR “LightGBM” OR “logistic regression” OR “Machine learning” OR “natural language” OR “natural language processing” OR “Natural language processing systems” OR “natural text” OR “natural text processing” OR “neural network” OR Neural networks OR “NLP” OR “random forest” OR “sentiment analysis “ OR “support vector machine” OR “support vector machines” OR “support vector network” OR “support vector networks” OR “support-vector machine” OR “support-vector machines” OR “support-vector network” OR “support-vector networks” OR “text modeling” ) OR AB ( Algorithm OR Algorithms OR “automated deception detection” OR “Automated text analysis” OR automation OR “Bidirectional Encoder Representations from Transformers” OR “classification algorithm” OR “deep learning” OR “feature extraction” OR “feature modeling” OR “k-Nearest Neighbor” OR “LightGBM” OR “logistic regression” OR “Machine learning” OR “natural language” OR “natural language processing” OR “Natural language processing systems” OR “natural text” OR “natural text processing” OR “neural network” OR Neural networks OR “NLP” OR “random forest” OR “sentiment analysis “ OR “support vector machine” OR “support vector machines” OR “support vector network” OR “support vector networks” OR “support-vector machine” OR “support-vector machines” OR “support-vector network” OR “support-vector networks” OR “text modeling” ) OR SU ( Algorithm OR Algorithms OR “automated deception detection” OR “Automated text analysis” OR automation OR “Bidirectional Encoder Representations from Transformers” OR “classification algorithm” OR “deep learning” OR “feature extraction” OR “feature modeling” OR “k-Nearest Neighbor” OR “LightGBM” OR “logistic regression” OR “Machine learning” OR “natural language” OR “natural language processing” OR “Natural language processing systems” OR “natural text” OR “natural text processing” OR “neural network” OR Neural networks OR “NLP” OR “random forest” OR “sentiment analysis “ OR “support vector machine” OR “support vector machines” OR “support vector network” OR “support vector networks” OR “support-vector machine” OR “support-vector machines” OR “support-vector network” OR “support-vector networks” OR “text modeling”)) | 17,652 |
| 4 | (TI ( internet OR online OR Platform OR “Social media” OR “Social network” OR “social networks” OR web ) OR AB ( internet OR online OR Platform OR “Social media” OR “Social network” OR “social networks” OR web ) OR SU ( internet OR online OR Platform OR “Social media” OR “Social network” OR “social networks” OR web )) | 107,951 |
| 5 | (TI(Dictionary OR Feature OR Features OR “Linguistic Inquiry and Word Count” OR LIWC OR “Named Entity Recognition” OR Semantic OR Sentiment OR “textual properties” OR “textual property” OR “Topic model” OR “Topic modeling” OR “topic modelling” OR “Word sketch” OR “word sketches” OR “word sketching”) OR AB(Dictionary OR Feature OR Features OR “Linguistic Inquiry and Word Count” OR LIWC OR “Named Entity Recognition” OR Semantic OR Sentiment OR “textual properties” OR “textual property” OR “Topic model” OR “Topic modeling” OR “topic modelling” OR “Word sketch” OR “word sketches” OR “word sketching”)) | 74,207 |
| 6 | 1 AND 2 AND 3 AND 4 AND 5 | 24 |
|  | Filter applied: Published Date 20120101-20221231 | 24 |
|  | Filter applied: Academic Journals | 22 |
