# Supplementary material for: Identifying Misinformation About Unproven Cancer Treatments on Social Media Using User-Friendly Linguistic Characteristics: Content Analysis
Source: JMIR Infodemiology. 2025 Feb 12;5:e62703. doi: 10.2196/62703 (PMC11888050; doi:10.2196/62703)
Supplement: Multimedia Appendix 2 [file infodemiology_v5i1e62703_app2.docx]

# Topic Name

1 714-X trimethylbicyclonitramineoheptane chloride

2 Actaea racemosa, black cohosh

3 Acupuncture

4 Alkaline diet

5 Aloe

6 Amygdalin, Laetrile

7 Andrographis paniculata

8 Angiostop

9 Anticancer psychotherapy

10 Antineoplaston therapy

11 antioxidants

12 Apitherapy

13 Applied kinesiology

14 Aromatherapy , fragrant substances, essential oils

15 Ashwagandha

16 Asparagus Extract

17 Aveloz , firestick plant, pencil tree, Euphorbia tirucalli

18 Ayurvedic medicine

19 Bach Flowers

20 Bioresonance therapy

21 Black Drawing Ointment

22 Breuss diet

23 Budwig protocol

24 Caesium chloride

25 Cancell

26 Cancer guided imagery

27 Cannabidiol

28 Cannabis

29 Cansema , black salve

30 Capsicum

31 Carctol

32 Cassava

33 Castor

34 CellAssure

35 Cevrogin Cholestrien

36 Chaparral

37 Chelation therapy, Cytokine therapy, Klehr's autologous tumor therapy, immunotherapy

38 Chelidonium

39 chiropractic

40 Chlorella

41 Clark's "Cure for All Cancers"

42 Coffee

43 Colloidal silver

44 Colon cleansing

45 Contreras therapy

46 Coral calcium

47 Craniosacral therapy , CST

48 Cupping

49 Cytotherapy

50 Dendritic Cell Therapy

51 DHEA (Dehydroepiandrosterone)

52 Di Bella Therapy

53 Dimethyl sulfoxide , DMSO

54 Ear candling

55 Echinacea

56 Electro Physiological Feedback Xrroid, Quantum Xrroid device

57 Electrohomeopathy , Mattei cancer cure

58 Ellagic acid

59 Emu oil

60 Essiac

61 Everything Herbs

62 Fasting

63 Fermented wheat germ extract (FWGE)

64 Freeda Vitamins

65 Ganoderma lucidum (Reishi mushroom)

66 Gc-MAF , Gc protein-derived macrophage activating factor

67 German New Medicine

68 Germanic

69 Gerson

70 Ginger

71 Ginseng

72 Glyconutrients

73 Goldenseal , Hydrastis canadensis

74 Gonzalez protocol

75 Gotu kola

76 Grapes

77 Greek cancer cure

78 Hallelujah diet

79 Hawk Dok Natural Salve

80 Healing Within Products & Services, Inc.

81 Herbalism, herbal remedies

82 Holistic medicine

83 Homeopath

84 Hoxsey therapy

85 Hydrazine sulfate

86 Hyperbaric oxygen therapy

87 Hypnosis

88 Immuno Boost Eximius

89 ImmunPro

90 Inonotus obliquus

91 Insulin potentiation therapy

92 Issels treatment

93 Juicing

94 Kelley treatment

95 kinesiology

96 Kombucha

97 Kousmine diet

98 Krebiozen

99 Krebiozen

100 Levodyn

101 Light therapy

102 Lipoic acid

103 Live blood analysis

104 Livingston-Wheeler Therapy

105 Livral Complex

106 Lorraine Day

107 Macrobiotic diet

108 magnetic

109 Mangosteen

110 McDougall diet

111 Meditation

112 Metabolic therapies

113 Milk thistle

114 Miracle Mineral Supplement

115 Mistletoe

116 Modified citrus pectin

117 Moerman Therapy

118 Moxibustion

119 Mushrooms

120 Native American healing

121 Nature's Treasure

122 Naturopathy

123 Nerium

124 Neuroimmunomodulation

125 Neuro-linguistic programming, NLP

126 Nieper therapy

127 Noni juice

128 nonotus obliquus , chaga mushroom

129 OliveLeafQi

130 Orgone , orgone accumulator

131 Orthomolecular medicine

132 Oxygen Health Systems

133 Oxygen therapy

134 Ozone

135 Pangamic acid

136 Pau d'arco

137 Phosphorylethanolamine

138 Polarity therapy

139 Poly-MVA

140 Pregnenolone

141 Protandim

142 Protandim

143 Psychic surgery

144 Pygeum

145 Qigong

146 Quercetin

147 Rauvolfia serpentina

148 Red clover , Trifolium pratense

149 Reiki

150 Revici's Guided Chemotherapy

151 Revivin

152 Rife Frequency Generator, radio waves

153 RIGVIR

154 Saw palmetto , Serenoa repens

155 Seasilver

156 Shark cartilage

157 Shiatsu

158 Sodium bicarbonate

159 Soursop

160 Stanley Brothers Social Enterprises

161 Strychnos nux-vomica , Ukrain

162 Sunstone

163 Superfood

164 The Vibrant Health Store

165 Therapeutic Touch

166 Trevinol ES Fibrin Defense Systemic Enzyme, Trevinol ES Health Joint & Inflammation Support, Trevinol Professional Blend

167 Uncaria tomentosa

168 Urine therapy

169 Venus flytrap

170 Vitacor

171 Vitalica

172 Vitamin C

173 Walnuts

174 Wheatgrass

175 Wild yam

176 Zoetron therapy
