# Supplementary material for: Identifying Misinformation About Unproven Cancer Treatments on Social Media Using User-Friendly Linguistic Characteristics: Content Analysis
Source: JMIR Infodemiology. 2025 Feb 12;5:e62703. doi: 10.2196/62703 (PMC11888050; doi:10.2196/62703)
Supplement: Multimedia Appendix 3 [file infodemiology_v5i1e62703_app3.docx]

# Appendix 3: Description of identified literature

Table S1. Description of the included articles

| **ID** | **Author name** | **Year** |  | **Goal of the study** | **Context** | Dataset sources | Dataset content | Data Size in the analysis |
| --- | --- | --- | --- | --- | --- | --- | --- | --- |
| 1 | Afroz | 2012 | [[147]](https://paperpile.com/c/FvocjX/jvUX4) | To detect stylistic deception. | Fiction writing | Extended-Brennan-Greenstadt corpus; Hemingway-Faulkner  Imitation corpus; Thomas-Amina Hoax corpus | Imitated writing samples; blog posts | 349 |
| 2 | Agarwal | 2022 | [[34]](https://paperpile.com/c/FvocjX/ulmBY) | To automatically identify fake news spreaders. | Fake news | PAN@CLEF 2020 | Tweets | 30,000 |
| 3 | Agarwal | 2022 | [[35]](https://paperpile.com/c/FvocjX/IHHx1) | To investigate the characteristics that make the news untrue and fake. | Fake news: politics | ISOT; Fake News Master; Liar; Kaggle; Emergent | News articles; claims | 52,431 |
| 4 | Agarwal | 2022 | [[122]](https://paperpile.com/c/FvocjX/XfLRv) | To improve the efficiency of fake news detection. | Fake news | PolitiFact, CREDBANK, BuzzFeed | Tweets; News articles; Social content | more than 37 million |
| 5 | Ahmad | 2020 | [[39]](https://paperpile.com/c/FvocjX/hlcC1) | To explore different textual properties that can be used to distinguish fake content from real. | Politics, entertainment, technology, and sports | ISOT; Kaggle; | News articles | 68,636 |
| 6 | Alkhodair | 2021 | [[36]](https://paperpile.com/c/FvocjX/ZvHiU) | To detect high-engaging breaking news and rumors in social media. | Fake news: 5 breaking news | PHEME | Tweets | 5,802 |
| 7 | Al-Rakhami | 2020 | [[124]](https://paperpile.com/c/FvocjX/ssTVm) | To analyze the credibility of information shared on Twitter pertaining the COVID-19 pandemic. | COVID-19 | Twitter API | Tweets | 409,484 |
| 8 | Alsmadi | 2020 | [[40]](https://paperpile.com/c/FvocjX/Z76hd) | To extract hybrid features from claims' content and metadata, enabling the automatic prediction of a claim's label (such as its truthfulness) | A wide range of contexts | Snopes, Politifact, and Emergent | News articles | 15,068 |
| 9 | Aneja | 2022 | [[41]](https://paperpile.com/c/FvocjX/AEpjl) | To identify linguistic characteristics of media news items that differentiate between fake and real news. | Fake news | Kaggle; Guardian; BuzzFeed; PolitiFact; FakeNews | News articles | 71,556 |
| 10 | Argyris | 2022 | [[156]](https://paperpile.com/c/FvocjX/ryCYI) | To identify sets of linguistic features that facilitate and inhibit the propagation of antivaccine-related content. | Antivaccine | Twitter API | Tweets | 4,668,980 |
| 11 | Basharat | 2018 | [[125]](https://paperpile.com/c/FvocjX/pWAq5) | To classify reliable and not-relaible user-generated data. | Fake news: Paris attacks;  India and West India;  Random tweets; 2016 | Twitter API | Tweets | 3,296 |
| 12 | Brasoveanu | 2021 | [[148]](https://paperpile.com/c/FvocjX/MLw4G) | To expand upon the idea of semantic fake news detection and look closely at two concepts: the ideas of credit score and degree of truthfulness. | Fake news: politics | LIAR dataset; Potlifact | Short political statements claims | 16,667 |
| 13 | Butt | 2021 | [[61]](https://paperpile.com/c/FvocjX/mwvNz) | To describe rumors and propose techniques to detect (or classify) rumors. | Fake news: nine news events | PHEME-9 | Tweets | 167,128 |
| 14 | Casillo | 2020 | [[62]](https://paperpile.com/c/FvocjX/QlnVS) | To develop a probabilistic approach to determining the degree of truthfulness of the information. | COVID-19 | LIAR, Credbank UNICO | Short political statement, tweets, and facebook posts | more than 60 million |
| 15 | Casillo | 2021 | [[158]](https://paperpile.com/c/FvocjX/yCkhS) | To develop fake news detection on English news article's title. | Fake news | Kaggle | News articles | 20,800 |
| 16 | Cerbin | 2021 | [[159]](https://paperpile.com/c/FvocjX/RjXId) | To identify those tweets that spread falsehoods regarding the use and effectiveness of masks in order to limit their appeal  and damage. | COVID-19 | Twitter API | Tweets | 4,042 |
| 17 | Choudhary | 2021 | [[65]](https://paperpile.com/c/FvocjX/7WPQF) | To detect and classify fake news using a linguistic model that detects language-driven features. | Fake news | FakeNewsData and Buzzfeed Political News | News articles | 502 |
| 18 | Choudhary | 2022 | [[66]](https://paperpile.com/c/FvocjX/QkeTK) | To propose a multi-task framework for fake news and rumor detection, predicting both the emotion and  the legitimacy of the text. | Wide range of contexts | PHEME 9; FakeNews AMT; Celeb; Gossipcop; | News articles; tweets | 117,354 |
| 19 | Dong | 2020 | [[191]](https://paperpile.com/c/FvocjX/lozRi) | To propose a hierarchical attention network with user and sentiment information (HiAN-US) for rumor detection. | Wide range of contexts | PHEME, Twitter 15 & 16 | Tweets | 8,594 |
| 20 | Fard | 2019 | [[42]](https://paperpile.com/c/FvocjX/0RSQ1) | To test a novel classiﬁcation approach, one-class classiﬁcation (OCC) of only rumors, without the non-rumor data points. | Wide range of contexts | Dataset developed by other researchers | Tweets | 147,335 |
| 21 | Fayaz | 2022 | [[196]](https://paperpile.com/c/FvocjX/ccgCB) | To test a random forest (RF) classifier for predicting fake or real news. | Politics, entertainment, technology, and sports | ISOT; Fake News Dataset | News articles | 44,919 |
| 22 | Felber | 2021 | [[67]](https://paperpile.com/c/FvocjX/Mu5Vh) | To classify COVID-19 related social media posts as either fake or real. | COVID-19 | Fake News Dataset | Tweets; Facebook, Instagram and other social media posts | 10,700 |
| 23 | Garg | 2022 | [[37]](https://paperpile.com/c/FvocjX/hZ0gn) | To apply linguistic features for detecting misinformation. | Fake news:politics | Random Political Dataset; Buzzfeed Political Dataset; McIntire Dataset  Reuter Dataset | News articles | 25,094 |
| 24 | Ghanem | 2020 | [[170]](https://paperpile.com/c/FvocjX/fg6wQ) | To detect fake news in Twitter at the account level using a neural recurrent model and a variety of different  semantic and stylistic features. | Fake news | Twitter API | Tweets accounts | 522,967 |
| 25 | Gravanis | 2019 | [[87]](https://paperpile.com/c/FvocjX/0Fa3y) | To test an enhanced linguistic feature set with word embeddings along with ensemble algorithms for classification of fake news. | Fake news | Kaggle-EXT; McIntire; BuzzFeed; Politifact; UNB | News articles | 33,076 |
| 26 | Hamidian | 2019 | [[113]](https://paperpile.com/c/FvocjX/GCooj) | To detect rumors as a type of misinformation propagation and to perform the task of rumor classification(RDC). | Fake news; rumors | Twitter API | Tweets | 18,118 |
| 27 | Hassan | 2020 | [[88]](https://paperpile.com/c/FvocjX/pGKou) | To detect the political fake statement by proposing two multi-stage feature-assisted neural models that consider claims and justifications as input in a stance detection manner. | Fake news: politics | LIAR dataset | Short political statements claims | 12,836 |
| 28 | Iwendi | 2022 | [[114]](https://paperpile.com/c/FvocjX/f5LlB) | To use the process of information fusion and obtain real and fake news data from news agencies and compare these against social media fake news | COVID-19 | Harvard Health Publishing, WHO, CDC, The New York Times; Facebook posts and  other medical sites | News articles; tweets | 2,264 |
| 29 | Jogalekar | 2020 | [[126]](https://paperpile.com/c/FvocjX/t10qb) | To detect rumors by using entity recognition and other text features | Fake news: 5 breaking news | PHEME | Tweets | 103,212 |
| 30 | Kalyanam | 2015 | [[127]](https://paperpile.com/c/FvocjX/hGxCZ) | To study the nature of misinformation and rumors in the United States during the fall of 2014 when Ebola cases were confirmed in North America. | Ebola virus | Twitter API | Tweets | 47 millions |
| 31 | Kasseropoulos | 2021 | [[161]](https://paperpile.com/c/FvocjX/ERCjx) | To test a style-based machine learning (ML) approach, which relies on manually extracted lexical features in the identification of fake news. | Fake news: famous people | Dataset developed by other researchers | News articles | 4,986 |
| 32 | Khan | 2022 | [[150]](https://paperpile.com/c/FvocjX/uXvWB) | To detect COVID-related fake news using feature extraction. | COVID-19 | Facebook; Twitter; The New York Times;  Harvard Health Publishing; WHO | News articles; tweets | 1,164 |
| 33 | Kumar | 2019 | [[151]](https://paperpile.com/c/FvocjX/i6LnW) | To develop an optimized learning model which classifies real-time tweets on the basis of truth value facilitating rumor  analysis. | Fake news: child-lifters in the Indian subcontinent | Twitter API | Tweets | 14,000 |
| 34 | Kumar Jain | 2020 | [[100]](https://paperpile.com/c/FvocjX/p1AAE) | To estimate a model that distinguishes fake news from a news article. | Fake news | FakeNewsNet McIntire Dataset | News articles | 6,755 |
| 35 | Kumari | 2021 | [[71]](https://paperpile.com/c/FvocjX/lkfHa) | To propose a deep multitask learning model that jointly performs novelty detection, emotion  recognition, sentiment prediction, and misinformation detection. | Wide range of contexts | Quora Question Pair; Unified; GoEmotion Datasets; ByteDance; Amazon product review; Fake News Challenge; Covid-Stance | Tweets; News articles; Reddit comments; other | more than 82 millinons |
| 36 | Kwon | 2013 | [[129]](https://paperpile.com/c/FvocjX/opc8i) | To identify characteristics of rumors by examining the following three aspects of diffusion: temporal,  structural, and linguistic. | Wide range of contexts | Dataset developed by other researchers | Tweets | not reported |
| 37 | Kwon | 2017 | [[72]](https://paperpile.com/c/FvocjX/FARdO) | To provide deep insight into the cumulative spreading patterns of rumors over time as well as at tracking the precise changes in predictive powers  across rumor features. | Wide range of contexts | Dataset developed by other researchers | Tweets | 192,359 |
| 38 | Li | 2022 | [[174]](https://paperpile.com/c/FvocjX/jATYV) | To identify different characteristics in fake and real news via behavioral and sentiment analyses and models based on feature elimination. | COVID-19 | Dataset developed by other researchers | Facebook; Instagram; tweets; public statements; press releases | 8,560 |
| 39 | Maan | 2022 | [[175]](https://paperpile.com/c/FvocjX/KiahM) | To detect rumors using stylometric and word vector features in machine learning models. | Wide range of contexts | Twitter-16 | Tweets | 818 |
| 40 | Mahbub | 2022 | [[57]](https://paperpile.com/c/FvocjX/hgICi) | To investigate the nature of a speciﬁc type of misinformation, i.e., rumors, surrounding COVID-19. | COVID-19 | Dataset developed by other researchers | News articles | 6,834 |
| 41 | Mazzeo | 2021 | [[130]](https://paperpile.com/c/FvocjX/ZvqIS) | To investigate the nature of a speciﬁc type of misinformation, i.e., rumors surrounding COVID-19. | COVID-19 | Search Engines: Google; Yahoo! | Meta titles; descriptions; URLs | 2,084 |
| 42 | Meel | 2021 | [[193]](https://paperpile.com/c/FvocjX/O3gMC) | To take leverage of the linguistic and stylometric information of annotated news articles. | Fake news | Kaggle | News Articles | 45,569 |
| 43 | Mondal | 2018 | [[152]](https://paperpile.com/c/FvocjX/aRVRl) | To identify tweets having high rumor probability. | Fake news: Chennai floods India | Twitter API | Tweets | 452,544 |
| 44 | Nikiforos | 2022 | [[194]](https://paperpile.com/c/FvocjX/4QR8e) | To introduce a well-defined method for fake news detection that uses both linguistic and network features. | Fake news: events in Hong Kong 2019 | Twitter API | Tweets | 2,366 |
| 45 | Niven | 2020 | [[58]](https://paperpile.com/c/FvocjX/GsWo0) | To classify whether a Twitter user has spread confirmed disinformation or not using two types of features thoughtfulness and emotional states | Fake news | PAN | Tweets | 30,000 |
| 46 | Octaviano | 2021 | [[108]](https://paperpile.com/c/FvocjX/yMG6Y) | To introduce two new features to indicate fake news: word bigram title and author published count. | Fake news | Kaggle | News Articles | 15,848 |
| 47 | Levi | 2019 | [[155]](https://paperpile.com/c/FvocjX/pFdPK) | To train a machine learning method using semantic representation, with a state-of-the-art contextual language model, and with linguistic features based on textual coherence metrics to distinguish between fake news and satire. | Fake news; satirical stories about politics | Dataset developed by other researchers | Tweets | 486 |
| 48 | Paschen | 2020 | [[195]](https://paperpile.com/c/FvocjX/0SysF) | To differentiate between fake and real news content based on their emotional appeals using AI. | Fake news | Dataset developed by other researchers | News articles | 150 |
| 49 | Patro | 2020 | [[90]](https://paperpile.com/c/FvocjX/b2UQp) | To predict the credibility of events based on the linguistic structure of Tweets about it. | Wide range of contexts | CREDBANK | Tweets | 66 million |
| 51 | Puraivan | 2021 | [[131]](https://paperpile.com/c/FvocjX/iQJxG) | To propose a framework for detecting fake news in online social networks through data mining techniques. | Fake news | Dataset developed by other researchers | Tweets | 2,366 |
| 52 | Raj | 2021 | [[177]](https://paperpile.com/c/FvocjX/2mgHg) | To identify rumors as soon as they appear online. | Fake news: nine news events | PHEME | Tweets | 6,425 |
| 53 | Rajabi | 2019 | [[74]](https://paperpile.com/c/FvocjX/pOcBd) | To identify vulnerable users for fake news mitigation using unsupervised learning with  the content-based, user-based, and network-based features. | Fake news | HOAXY | Tweets | 20,987,210 |
| 54 | Rastogi | 2022 | [[95]](https://paperpile.com/c/FvocjX/Qxqyr) | To distinguish real and satirical news in the model that combines style based and propagation-based features. | COVID-19 | Fak_Cov | Tweets | 3,000 |
| 55 | Reddy | 2020 | [[165]](https://paperpile.com/c/FvocjX/yK3o3) | To improve identifying fake news on social media with linguistic features without using any other related metadata | Fake news | FakeNewsNet; McIntire Dataset | News articles | 6,756 |
| 56 | Rezayi | 2021 | [[178]](https://paperpile.com/c/FvocjX/pXDRE) | To build a novel multimodal classifier using deep neural network that incorporates relaying features, textual features,  and network feature concatenated with each other in a late fusion manner | Fake news: Brussels attacks 2016 | Dataset developed by other researchers; PHEME | Tweets | 100,123 |
| 57 | Anggrainingsih | 2022 | [[166]](https://paperpile.com/c/FvocjX/sTcHX) | To identify rumors on Twitter by utilizing sentence embedding using Bidirectional Encoder Representations from Transformers BERT | Fake news: nine news events | PHEME | Tweets | 5,791 |
| 58 | Sahana | 2015 | [[134]](https://paperpile.com/c/FvocjX/j1FzY) | To automatically detect the rumors spreading on Twitter and identify their source. | Fake news: London riots 2011 | Twitter API | Tweets | 2,085 |
| 59 | Saikh | 2019 | [[84]](https://paperpile.com/c/FvocjX/xlw9Q) | To identify fake news through stance detection. | Fake news | Fake News Challenge FNC-I | News articles | 75,385 |
| 60 | Sailunaz | 2022 | [[136]](https://paperpile.com/c/FvocjX/sbhXV) | To classify rumor and non- rumor tweets by applying a novel tweet and user feature ranking approach. | Fake news: nine news events | PHEME | Tweets | 5,802 |
| 61 | Sandrilla | 2022 | [[75]](https://paperpile.com/c/FvocjX/5IxWG) | To create a standardized method of accurately measuring reliability of information posted on social media. | Fake news | Kaggle | News Articles | 23,000 |
| 62 | Seddari | 2022 | [[76]](https://paperpile.com/c/FvocjX/VXnfP) | To establish a hybrid linguistic and knowledge-based fake news detection system that combines linguistic features and a set of knowledge-based features. | Fake news: election | Buzzfeed Political News | Facebook | not reported |
| 63 | Lofti | 2021 | [[102]](https://paperpile.com/c/FvocjX/pbtp7) | To identify new structural features of rumor conversations on Twitter and use it to develop a computational model for detecting rumor-based conversations. | Fake news: 5 breaking news | PHEME | Tweets | 5,802 |
| 64 | Sharma | 2022 | [[138]](https://paperpile.com/c/FvocjX/bl30P) | To identify important features to distinguish rumor-spreading tweets from authentic tweets. | Wide range of contexts | PHEME, Twitter15 & Twitter16 | Tweets | 105,354 (not fully reported) |
| 65 | Sharma | 2022 | [[180]](https://paperpile.com/c/FvocjX/hYVH4) | To propose a framework utilizing 17 linguistic features of text for fake news identification. | Fake news: politics | LIAR dataset | Short political statements claims | 12,837 |
| 66 | Sharma | 2019 | [[139]](https://paperpile.com/c/FvocjX/gbViW) | To identify the features that can be helpful in predicting whether a given Tweet is rumor or information. | Fake news: nine news events | PHEME | Tweets | 5,802 |
| 67 | Shelke | 2022 | [[140]](https://paperpile.com/c/FvocjX/t0ZzY) | To detect misinformation employing user-based, content-based, lexical-based features and post sequences. | Fake news: events discussed on Twitter | Twitter API | Tweets | 267,708 |
| 68 | Shelke | 2021 | [[182]](https://paperpile.com/c/FvocjX/P8j6e) | To identify various features in rumor detection in social networks. | Fake news | Twitter API | Tweets | 268,199 |
| 69 | Tafannum | 2023 | [[137]](https://paperpile.com/c/FvocjX/voth9) | To illustrate which feature of the data contributes the most for a post to have been predicted as a rumor or a non-rumor | COVID-19 and other topics | Twitter 15 & 16; Constraint@AAAI2021 COVID19 Fake News Detection | Tweets | 10,296 |
| 70 | Sicilia | 2017 | [[47]](https://paperpile.com/c/FvocjX/AnC1l) | To present a novel rumour detection system and identify health-related rumours on Twitter. | Zika virus | Twitter API | Tweets | 800 |
| 71 | Singh | 2020 | [[77]](https://paperpile.com/c/FvocjX/y9pSI) | To automatically distinguish between rumors and non-rumors on Twitter using linguistic features. | Fake news: nine news events | PHEME | Tweets | 5,802 |
| 72 | Singh | 2019 | [[183]](https://paperpile.com/c/FvocjX/s1s6s) | To compare machine learning classifiers with deep Learning models using Recurrent Neural Networks for rumor classification. | Fake news: nine news events | PHEME | Tweets | 5,802 |
| 73 | Slimi | 2019 | [[79]](https://paperpile.com/c/FvocjX/6JL4Z) | To determine URL content credibility by measuring how frequently it is shared during a Twitter event. | Wide range of contexts | CREDBANK | Tweets | 50,000 |
| 74 | Sotirakou | 2021 | [[110]](https://paperpile.com/c/FvocjX/IkAA7) | To build a detection tool for online disinformation campaigns by examining content-based features related to language use, emotions, and engagement features through explainable machine learning. | Fake news | Made their own | Facebook posts, news articles | 33,806 |
| 75 | Sun | 2019 | [[167]](https://paperpile.com/c/FvocjX/rdN5z) | To identify a machine learning classifier that determines the validity of news based on the word distributions and specific linguistic and stylistic differences of the first few sentences of an article | Fake news | Made their own; Fake News Codex | News articles | 2,107 |
| 76 | Tuarob | 2014 | [[49]](https://paperpile.com/c/FvocjX/o8mPf) | To address the limitations posed by the traditional bag-of-word-based methods by proposing heterogeneous features in combination with ensemble machine learning techniques to discover health-related information. | Wide range of contexts | Twitter API; Facebook API | Tweets; Facebook posts | more than 700 million |
| 77 | Vadavalli | 2021 | [[83]](https://paperpile.com/c/FvocjX/nCnF7) | To classify Tweets as true or false and classify Twitter users as reliable or unreliable using automatic feature extraction technique along with a feed-forward neural network classification system. | Wide range of contexts | My Information Bubble (MIB) | Tweets | 16964 (not fully reported) |
| 78 | Van de Guchte | 2020 | [[50]](https://paperpile.com/c/FvocjX/TPpbn) | To construct a deep learning model that combines a comprehensive set of network and linguistic features to classify articles as misinformation or trusted information. | Fake news: politics | Twitter API | Articles on Tweets | 1,300 |
| 79 | Vereshchaka | 2020 | [[196]](https://paperpile.com/c/FvocjX/huIuq) | To identify fake news by analyzing fake news features and identifying the textual and sociocultural characteristics fake news features. | Fake news | FakeNewsNet | News articles | 2,787 |
| 80 | Verma | 2021 | [[81]](https://paperpile.com/c/FvocjX/Zdznq) | To propose a two-phase benchmark model named WELFake based on word embedding (WE) over linguistic features for fake news detection using machine learning classification. | Fake news | Benjamin; Burfoot; Buzzfeed; Credbank; Fake News challenge; FakeNewsNet; LIAR; Reuters; McINtire & Kaggle | News articles; tweets | 72,134 |
| 81 | Vijeev | 2018 | [[51]](https://paperpile.com/c/FvocjX/sJCh2) | To compare 3 different machine learning models, each model using only user, only content, and also a hybrid of these features to identify fake news. | Fake news: nine news events | PHEME | Tweets | 29,010 |
| 82 | Volkova | 2018 | [[97]](https://paperpile.com/c/FvocjX/rX9o3) | To gain deeper insights into writers’ intent behind digital misinformation by analyzing psycholinguistic signals: moral foundations and connotations extracted from different types of deceptive news ranging from strategic disinformation to propaganda and hoaxes. | Wide range of contexts | Made their own | Disinformation on news sites; propaganda pages; tweets | 27,827 |
| 83 | Volkova | 2017 | [[104]](https://paperpile.com/c/FvocjX/cp1Fx) | To build linguistically-infused neural network models that jointly learn from tweet content and social network interactions to classify suspicious and verified news tweets and infer specific types of suspicious news. | Fake news: Brussels attacks 2016 | Twitter API | Tweets | 133,848 |
| 84 | Xuan | 2019 | [[99]](https://paperpile.com/c/FvocjX/pATCV) | To identify 18 features to detect misinformation on social media. | Fake news: eight emergency events | RumorEval | Tweets | 11,136 |
| 85 | Zhou | 2020 | [[106]](https://paperpile.com/c/FvocjX/rCPjF) | To investigate news content at various levels: lexicon-level, syntax-level, semantic-level, and discourse-level. | Fake news:politics | Buzzfeed; Politifact | News articles; fake news stories | 1265064 |
| 86 | Purevdagva | 2020 | [[197]](https://paperpile.com/c/FvocjX/BnnZ2) | To detect fake political speech using different classification methods for extracting features including speech subject, location,  speaker’s profile, speaker’s credibility, and speech context information. | Fake speech: politics | LIAR dataset | Short political statements claims | 12,791 |
| 87 | Wang | 2018 | [[184]](https://paperpile.com/c/FvocjX/zGpxk) | To capture shades of truth (truth, mostly truth, half-truth, lie) in a systematic exploration of a variety of signals from both news and social media, and give an  analysis of the underlying features. | Wide range of contexts | Made their own (Twitter API, PolitiFact; The Onion; American News; Natural News; and BBC News ) | Articles, tweets, statements | more than 1 million |
| 88 | Kula | 2020 | [[82]](https://paperpile.com/c/FvocjX/U9Ftq) | To evaluate neural network-based approach to text analysis and fake news detection | Fake news | ISOT Fake News Dataset; Getting real about fake news | News Articles | 57,301 |
| 89 | Gautam | 2020 | [[56]](https://paperpile.com/c/FvocjX/sVZy0) | To test robust yet simple fake news detection system, leveraging the tools for paraphrasing, grammar-checking, and word-embedding. | Fake news: seven domains | Celebrity; FakeNews AMT | News Articles | 980 |

**References**

[Not included in the main reference list]

191. [Dong S, Qian Z, Li P, Zhu X, Zhu Q. Rumor detection on hierarchical attention network with user and sentiment information. Natural Language Processing and Chinese Computing. Cham: Springer International Publishing; 2020. pp. 366–377.](http://paperpile.com/b/FvocjX/lozRi)

192. [Fayaz M, Khan A, Bilal M, Khan S. Machine learning fake news classification with optimal feature selection. Research Square. 2021. doi:](http://paperpile.com/b/FvocjX/ccgCB)[10.21203/rs.3.rs-835344/v1](http://dx.doi.org/10.21203/rs.3.rs-835344/v1)

193. [Meel P, Vishwakarma DK. A temporal ensembling based semi-supervised ConvNet for the detection of fake news articles. Expert Syst Appl. 2021;177: 115002.](http://paperpile.com/b/FvocjX/O3gMC)

194. [Nikiforos MN, Vergis S, Stylidou A, Augoustis N, Kermanidis KL, Maragoudakis M. Fake news detection regarding the Hong Kong events from tweets. Artificial Intelligence Applications and Innovations AIAI 2020 IFIP WG 125 International Workshops. Cham: Springer International Publishing; 2020. pp. 177–186.](http://paperpile.com/b/FvocjX/4QR8e)

195. [Paschen J. Investigating the emotional appeal of fake news using artificial intelligence and human contributions. Journal of Product & Brand Management. 2019;29: 223–233.](http://paperpile.com/b/FvocjX/0SysF)

196. [Vereshchaka A, Cosimini S, Dong W. Analyzing and distinguishing fake and real news to mitigate the problem of disinformation. Comput Math Organ Theory. 2020;26: 350–364.](http://paperpile.com/b/FvocjX/huIuq)

197. [Purevdagva C, Zhao R, Huang P-C, Mahoney W. A machine-learning based framework for detection of fake political speech. 2020 IEEE 14th International Conference on Big Data Science and Engineering (BigDataSE). IEEE; 2020. doi:](http://paperpile.com/b/FvocjX/BnnZ2)[10.1109/bigdatase50710.2020.00019](http://dx.doi.org/10.1109/bigdatase50710.2020.00019)
